# Portraying accent stereotyping by second language speakers

**DOI:** 10.1371/journal.pone.0287172

**Published:** 2023-06-15

**Authors:** Yizhou Lan, Tongtong Xie, Albert Lee

**Affiliations:** 1 School of Foreign Languages, Shenzhen University, Shenzhen, China; 2 Department of Linguistics, The Education University of Hong Kong, Tai Po, Hong Kong SAR; Hong Kong Polytechnic University, HONG KONG

## Abstract

Stereotyping towards the second language accent of second language learners is extensively seen even when the content of learner speech can be understood. Previous studies reported conflicting results on accent perception by speakers of second languages, especially among homogenous learners. In this paper, we conducted a survey and two experiments to test whether Mandarin-speaking advanced learners of English may give harsher accent ratings to their fellow learners than to Standard American English speakers. The survey was designed to understand the L2 listeners’ beliefs about accented speech. In Experiment 1, participants rated short audio recordings of L2 learner’ and Standard American English speech; in Experiment 2, they did the same in a more detailed word-in-sentence accent rating task. Results showed a markedly high level of perceived L2 accentedness for several learner speech stimuli despite good intelligibility, especially for the strongly-accented Cantonese passage and for specific vowel and consonant types. The findings reveal the existence of native-speakerism in China and highlight existing accent stereotypes. Implications for policymaking and language teaching are discussed.

## Introduction

### Background

The evaluation of second language (L2) accents has long been a purely linguistic topic, but the listeners’ *beliefs* about such were less explored [1]. Such beliefs may come from speakers of the inner circle varieties of English (primarily those from the UK, North America, Australia, and New Zealand) [2] or L2 speakers themselves [2], and can be more often than not inaccurate, biased or overgeneralized. When these beliefs translate into discrimination [3], a series of adverse social issues may arise, such as adolescent depression, work stress, discrimination in employment, and harassment based on the L2 accent [4–8]. Following previous work on language attitudes such as [9, p.194], here we define L2 accent beliefs as “the listener’s prejudice, stereotypes, and beliefs formed to make evaluations on or inferences about the L2 speaker’s communication skills, personality, or social status.” In this paper, we present a set of accent rating experiments and explore how Chinese learners of English judge their own variety as well as Standard American English.

### From native-speakerism to world Englishes

Stereotypes of L2 pronunciation varieties are often associated with the concept of “native-speakerism”. Inner circle English speakers that reflect a region’s relatively affluent middle class were often considered the best models and teachers [10]. It is the ideology that “native speakers” are the best models and teachers of English because they represent a “Western culture” from which spring the ideals both of English and of the methodology for teaching it [10]. As the definition of a “native language” may lead to undesired discrimination in sociolinguistic research [11], here we define what was usually regarded the ‘Native English accent’ in this study as the Standard American Accent. According to He and Li [12], 79% of Chinese interviewees in their study expressed a clear preference for American English as opposed to British.

Accompanying this ideology is the assumption that inner circle varieties of English are more intelligible [13], which is central to successful communication. In everyday settings, native-speakerism translates into the view that inner-circle accent varieties are better than L2 ones, leading to accent stereotypes. One example is Raisler [14]. In their study, 730 American students regarded an unseen speaker with an apparent L2 accent as less interesting, less convincing, and even less physically attractive than an inner circle English speaker.

Along with the emerging notion of World Englishes [15], accumulating evidence shows that L2 learner’s varieties of English can be easily understood in international communication, and more people are accepting them. For example, British-accented English speakers rated Cantonese-accented English as highly intelligible and its speakers as intelligent and likable [13]. Today, most English language users are non-native speakers, and they use English as a means of communication [16]. These lingua franca users are considered highly effective intercultural communicators [17]. This gradual shift away from native-speakerism thus calls for more research on the discrimination situation against L2 speakers today. Therefore, as the number of L2 English learners increases globally, accent stereotyping may become more prominent in magnitude. Here we explore this question with Mandarin-speaking L2 learners as a representative test case.

### Measuring bias toward an accent

Although Chinese learners constitute the largest population of English learners globally [18, 19], and in turn, speak an important variety of world Englishes, it does not follow that they would necessarily hold a positive attitude towards their own English accent. While previous studies have reported that L2 listeners tend to find it easier to understand L2 speakers of the same L1 background [20, 21] and that English with a strong Mandarin accent is fully and easily understandable to British or Standard American English listeners [22], whether Chinese learners will regard this familiar accent as more understandable than other unfamiliar varieties remains an empirical question.

L2 or foreign language accent perception is usually analyzed from three perspectives: intelligibility, the actual understanding of the incoming phonetic information; comprehensibility, the perceived ease or difficulty of understanding; and accentedness, the perceived degree of strength of accent [23]. In some cases, L2 learners with an accent stereotype may tend to perceive their accent variety as more accented than it actually is. As an indicator of such situations, the discrepancy between intelligibility and comprehensibility towards that variety can be large [24]. Accentedness is the subjective appraisal of how positively or negatively one evaluates the speech, but intelligibility reflects objective understanding [22–25]. For example, according to Brennan and Brennan [26], listeners’ negative attitudes towards Mexican English speakers were significantly correlated to high accentedness ratings on their English speech. While actual intelligibility of accented speech is closely related to the type, severity, and frequency of divergences from the norms [23, 25, 27], the perception of accent is influenced primarily by social factors [28–30].

Studies from as early as Chang [31] to more recent ones as Deterding [32] have pinpointed specific non-standard productions by Mandarin speakers of English, such as those involving the consonants [ʃ, θ, ð, h, l, r, w, v, pr, tr, kr]; the vowels [ɪ, æ, ʊ, u, ə]; tonal transfer on stress, to name just a few. However, these specific L2-colored productions have not been examined for listener intelligibility and perceived accentedness by Chinese ESL learners in previous studies.

### Research questions and hypotheses

Our overarching research question is whether Chinese Mandarin-speaking learners of English evaluate their own accents more comprehensible and less accented than Standard American English and Cantonese-accented English. We are also interested in how Mandarin- and Cantonese-accented stimuli with different degrees of accent strength are evaluated.

We hypothesized that (H1: intelligibility) Mandarin-accented English would yield the highest intelligibility scores among the three language groups. Likewise, we also tested the hypothesis that (H2: comprehensibility) Mandarin-accented English would score the lowest on comprehensibility. In addition, we tested the working hypothesis that (H3: accentedness) participants would rate Mandarin-accented English as more accented than the Cantonese and Standard American English stimuli.

H1 is based on previous findings that L2 listeners tended to find it easier to understand L2 speakers of the same L1 background compared to unfamiliar ones [22, 23]. As H2 is related to participants’ subjective perception of the ease of understanding, we speculated that they would be more hostile towards Mandarin- and Cantonese-accented English than the Standard American variety. Testing H3, especially compared with the scores of Cantonese-accented English, will shed new light on our research question. Moreover, we also lay out strength-wise predictions for H1-H3 (Table 1).

**Table 1:** Summaries of Experiment 1 Hypotheses. H1: intelligibility, H2: comprehensibility, H3: accentedness, H4: bias towards L2 accents, H5: individual sounds, each with overall and language-specific predictions.

Predictions	Overall Predictions	Mandarin Accent Strengths	Cantonese Accent Strengths
Hypotheses			
H1: Intelligibility	Mandarin–highest	Strong–highest	Strong–lowest
H2: Comprehensibility	Mandarin–lowest	Strong–lowest	Strong–lowest
H3: Accentedness	Mandarin–highest	Strong–highest	Strong–highest
H4: |Accentedness-Intelligibility|	Mandarin and Cantonese–higher than English	All strengths–higher than English	All strengths–higher than English
H5: Individual Sounds	Difficult sound–larger accentedness, smaller intelligibility	All strengths–Difficult sound has larger accentedness and smaller intelligibility	All strengths–Difficult sound has larger accentedness and smaller intelligibility

Since previous studies suggest that L2 speakers may be overtly self-aware of the L2-accented speech, regarding that as poorly accented, we hypothesized H4 that the scores of intelligibility and accentedness should both be very high if there is a stereotypical attitude toward the L2 accent. Consequently, the differences between accentedness and intelligibility scores for Cantonese and Mandarin speeches will be smaller than that of Standard American English speech (H4: bias towards L2 accents). H1 –H4 were tested with Experiment 1 (further described in ‘Methods’).

In addition, in terms of fine-grained accent perception, we are also interested in whether specific segments might lead to higher perceived accentedness ratings or lower intelligibility. Based on predictions by Flege et al. [25], that L2 sounds which are “phonetically relevant” (p. 3133), i.e. challenging to learners, usually depict accented speech; whereas less challenging sounds enable learners to establish a new category and therefore show less accent. Therefore, we hypothesized that (H5: individual sounds) learner productions of words containing the challenging consonants /ʃ, θ, ð, h, l, r, w, v, pr, tr, kr/ and the challenging vowels /ɪ, æ, ʊ, u, ə/ will be rated more accented and less intelligible than mono-syllabic words containing /b, k, g, t, d/ and /oʊ/, which exist in both Mandarin and American English. We tested H5 with Experiment 2. To avoid potential confounds, fine-grained perceptions of Cantonese-accented English are not pursued here. Table 1 shows an overview of the predictions of H1-H5.

Our study is the first recent empirical study to test the language attitudes towards varieties of Standard American, Mandarin and Mainland Cantonese accents of Englishes and the first study to question Chinese L2 English speakers’ language attitude towards their own variety and other regional varieties through the lens of L2 English speech. It is also among one of the few studies joining a holistic accent perception task and a more specific one including individual sounds.

## Materials and methods

A mixed research design including qualitative and quantitative elements was employed. There are three components: a set of survey questions and two experiments containing comprehension questions, identification tasks, and rating tasks. The two experiments (Experiments 1 and 2 henceforth) followed Evans and Iverson’s [33] paradigm of testing overall and fine-grained segmental accent perception using paragraph and word stimuli, respectively. The design of this study has been approved by the first authors’ Institutional Review Board of the School of Medicine, Shenzhen University. The study was conducted according to the principles expressed in the Declaration of Helsinki. Informed written consent (as outlined in the PLOS consent form) was obtained from all participants.

### Participants

Fifty-four Chinese college students (23 female) from a top university in the Guangdong Province, China, participated in a survey and Experiment 1. Eighty-seven (29 female) students, including the previous 54, participated in Experiment 2 (*N* = 87). The average age of participants was 18.7 in Experiment 1 (*SD* = 0.69) and 18.9 in Experiment 2 (*SD =* 0.77). All participants spoke Putonghua (i.e., Standard Mainland Mandarin) as L1, and 11 (12.6% of Experiment 2 participants) also spoke the regional language Cantonese. None of them spoke other regional languages. All of them had studied English as their only foreign language for an average of 11.8 years of schooling (*SD =* 2.06). They could be collectively described as advanced learners of English, with English scores on the National College Entrance Examination ranging between 95 and 141 out of 150, with an average of 125.2 (*SD =* 12.37). Participants indicated that they were not trained with accent detection skills and had never participated in a similar study before.

#### Survey

The questionnaire items were adapted from the Speech Evaluation Instrument (SEI) [2,9] and the Attitude Towards Language Scale (AToL) [30]. Each participant was presented with the following prompt questions in Chinese. Q2 and Q3 were open-ended questions, whereas Q1, Q4, Q5 and Q6 required rating responses on a Likert scale (1–5). All responses were written in Chinese. After giving responses, participants could add more comments if they wished. The questions are as shown below. All survey responses were translated into English for grouping and analysis. We coded Q2 by the number of mentions of English accents, and Q3 by sorting positive and negative comments on “non-mainstream” regional accents into three topics, i.e., enunciation and fluency, prosody and emotive evaluations. Q1 “Self-satisfaction” and Q6 “Equal” were subsequently included as fixed factors in the statistical model for Experiment 1.

**Table:** 

Q1	What do you think of your own accent of speaking English?
	To what extent are you satisfied with it? (1 = not satisfied at all; 5 = fully satisfied)
Q2	What accents of the English language are you familiar with?
Q3	What accent-related comments have you or other regional accent speakers received?
Q4	What is your goal or expectation in your English pronunciation? (1 = retain Chinese accent; 5 = become “native-like” in English)
Q5	To what extent do you think your accent may reflect your English ability? (1 = irrelevant; 5 = relevant)
Q6	What is your opinion on the idea that all accents are equal in status? (1 = totally agree; 5 = totally disagree)

#### Experiment 1

Experiment 1 is an accent judgment task using stimuli of learner speech categorized as having strong, moderate, and weak accent strengths [34, 35]. Stimuli were initially 10 Mandarin and Cantonese-accented, phonetically balanced passages from the Speech Accent Archive (https://accent.gmu.edu/). In addition to Mandarin, we added Cantonese, an unfamiliar regional language to Mandarin speakers, for a better perspective of participants’ attitudes towards Cantonese- and Mandarin-accented Englishes. The stimuli passage is presented as follows.

“*Please call Stella*. *Ask her to bring these things with her from the store*: *Six spoons of fresh snow peas*, *five thick slabs of blue cheese*, *and maybe a snack for her brother Bob*. *We also need a small plastic snake and a big toy frog for the kids*. *She can scoop these things into three red bags*, *and we will go meet her Wednesday at the train station*.”

Productions from the *Speech Accent Archive* were normalized for amplitude and duration and then categorized by two phonetically trained Standard American-accented English-speaking judges into three levels of strength: strong-accent, moderate-accent, and weak-accent. The reason for using a three-level categorization is because we intended to give a more fine-grained portrait of the learners’ language attitudes in their own perception. Only making a holistic distinction on whether certain accents “exist” is not enough to elicit accurate accent perceptions. The two judges agreed on the categorization of accent strengths for all items, but each expressed a lack of confidence for one item (Cronbach’s α = .886). The two excerpts that the two judges did not reach a confident consensus on were discarded. One sentence with Standard American English, the most accepted variety of English pronunciation by Chinese students [12], was set as a control stimulus. Finally, eight sentence excerpts are presented to listeners in randomized order. The inter-stimulus interval (ISI) was 30 seconds.

#### Experiment 2

Experiment 2 is also an accent judgment task, with stimuli from Mandarin-accented utterances retrieved from *The Linguistic Data Consortium* (www.ldc.upenn.edu). The stimuli were manipulated in Praat [36] to normalize amplitude and duration. The choice of individual stimuli was based on common pronunciation errors by Mandarin speakers [31, 32]. We chose mono-syllabic and disyllabic words containing the consonants /ʃ, h, θ, ð, l, r, w, v, pr, tr, kr/ and the vowels /ɪ, æ, ʊ, u, ə/. The target word productions were spliced into the same utterance “Now I say _____ again.” Words with initial /b, k, g, t, d/ and the vowel /oʊ/ were added as control sounds. Frequently used words were chosen to ensure the accessibility of stimuli to participants except for a few phonetic contrasts where a simpler word is not available, so we added a few more complex words (e.g., *villa*, *trolley*, *troop*, *whack*) instead for convenience. To ensure that these words can be correctly recognized by the participants and to avoid the effect of familiarity at the same time, we checked if their peers can pronounce the list of these words properly beforehand. All the words were correctly pronounced. In total, 42 words × 2 repetitions = 84 sentence tokens were randomized and compiled into one audio file with an ISI of 6 seconds between each sentence. Participants were given a 3-minute break halfway into this experiment. Table 2 presents the complete list of the stimuli. Dictation, the usual method for eliciting intelligibility [37, 38], was replaced by a forced-choice word recognition task (e.g., choosing between *hit*, *sit* or between *thing*, *sing*).

**Table 2:** Stimuli used in Experiment 2.

Vowel types	1. AmE /ɪ/	2. AmE /æ/	3. AmE /ʊ, u/	4. AmE /ə/	5. AmE /oʊ/
Cons. Types					
**1. /ʃ, h/**	hit, ship	Hat	shoes	her	hole
**2. /θ, ð/**	thing, this	thank, that	through	the	though
**3. /w, v/**	villa	whack	wood	however	vote
**4. /l, r/**	live	lack, rat	rude	ruler	low, row
**5. /pr, tr, kr/**	print, trolley	track, crack	cruel, troop	proper	crow
**6. /b, k, g, t, d/**	kid, tip	cat, tap	book, took	collect, today	goat, toe

### Procedure

In Experiment 1, participants sitting in a computer room with HD headphones listened to the excerpts. They were instructed to provide intelligibility, comprehensibility, and perceived accentedness scores (S1 Appendix). All scores were obtained through an online survey system (www.wjx.com). Participants provided intelligibility scores by completing a comprehension test (on a scale of 0–4; 4 indicating providing four correct answers to the four questions randomly selected from the following: “Who was called? What was bought from the store? What was bought for Bob? What were bought for the kids? How many red bags were there? When were they going to meet? Where were they going to meet?” Each question contained four choices, among which only one was correct). They also gave comprehensibility and accentedness scores by rating statements such as “I don’t have to make an effort to understand the excerpt,” and “the excerpt is heavily accented,” respectively, on a Likert scale from 1 (strongly disagree) to 5 (strongly agree). All three indicators were subsequently aligned to a 1–5 scale.

In Experiment 2, participants heard pre-recorded stimuli sentences and at the same time saw three words on a screen: one of them was the correct target word (e.g., *think*), and the other two were minimal-pair distractors (e.g., *sink*, *thank*). For intelligibility, participants were asked to click on words they felt they heard. After they finished clicking, participants were required to rate the perceived accentedness of the token. We subsequently coded the incorrect responses as 0 and correct ones as 1. They also gave accentedness ratings on Likert scales of 1 (unaccented) -5 (very heavily accented) for the question, “To what extent do you think the speech is accented?” To avoid confusion, intelligibility scores and accentedness ratings were presented in the inverse: a better-perceived stimulus entails a greater intelligibility and a smaller accentedness rating.

## Results

### Survey

The 54 valid responses to Q2 and Q3 are presented in the S1 Table. For Q2 (What accents of the English language are you familiar with?), 44 participants shared at least three accents worldwide, whereas only 10 reported less than 2. The most commonly mentioned accents were British English (39 participants), American English (32 participants) and Chinese English accents (29 participants). For Q3 (What accent-related comments have you or other regional accent speakers received?), only one participant reported a positive comment stating that the Chinese regional accents offer “a close feeling of friendliness and benevolence”. The other 53 participants reported at least one negative comment. No neutral comment was stated. Theme-wise, 27 participants reported concerns about enunciation and fluency, 17 on prosodic unnaturalness such as errors of stress, rhythm, and intonation, and 17 offered negative emotive evaluations or inferences on the social status of the speaker.

A good knowledge of English accents worldwide was seen from responses to Q2, with 44 (81.5%) out of 54 participants mentioning at least three global English accents. Q3 yielded statements of actual accent stereotyping. In response to Q3, 22 participants (40.7%) commented that Chinese “non-mainstream” regional accents of English seemed “non-standard”, and 20 participants (37.0%) mentioned “unnaturalness”. A few participants commented on the northeastern Chinese regional accent, which sounded “funny” and “corny”.

Q1 (What do you think of your own accent of speaking English?) yielded a moderate average for self-evaluation (*M* = 2.88, *SD =* 0.45). Q4 (What is your goal or expectation in your English pronunciation?) yielded relatively low ratings (*M* = 1.81, *SD =* 0.44), showing that most participants favored their own English pronunciation. Higher ratings (*M* = 3.83, *SD =* 0.72) on Q5 (To what extent do you think your accent may reflect your English ability?) suggest that learners were prone to regard pronunciation as an important element in overall English ability. The moderately high ratings (*M* = 3.30, *SD =* 1.13) on Q6 (What is your opinion on the idea that all accents are equal in status?) indicate that the participants did prefer some accent varieties over others. The interplay of responses to Q4-6 was tested by Pearson’s correlations. The correlations between Q4 and Q5 and Q4 and Q6 were very low and not significant (*r*(53) = .02, *p* > .05; *r*(53) = .04, *p* > .05), but the correlation between Q5 and Q6 was low and weakly significant (*r*(53) = .32, *p* < .05), showing that those with an accent bias were slightly more likely to consider accent to be an important aspect of English learning.

### Experiment 1

**Fig 1:**
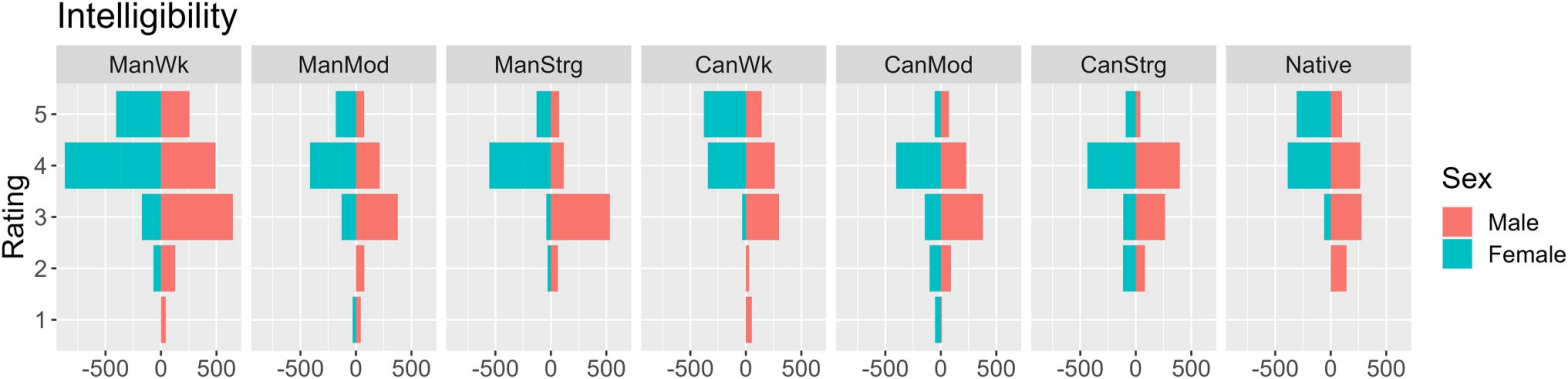
Distribution of intelligibility ratings by speaker accent (y-axis) and participants’ gender (bar colours).

**Fig 2:**
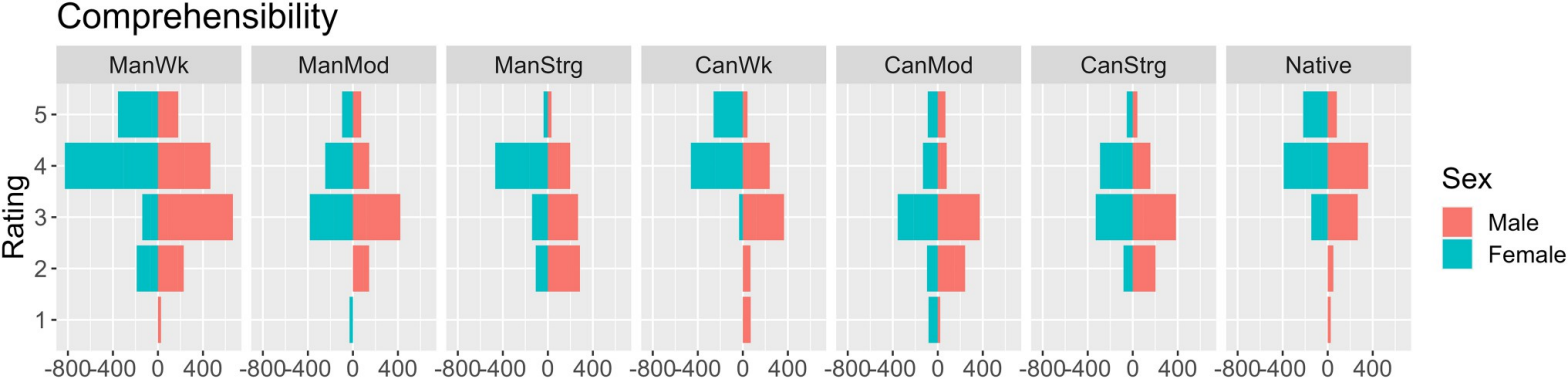
Distribution of comprehensibility ratings by speaker accent and participants’ gender.

**Fig 3:**
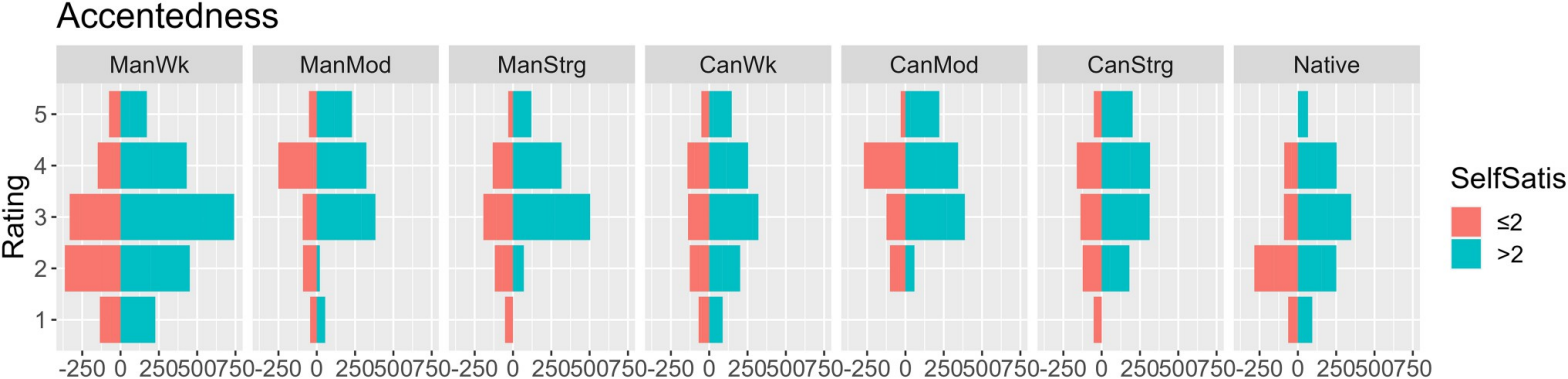
Distribution of accentedness ratings by speaker accent and self-satisfaction.

Fifty-four participants provided intelligibility (Fig 1) scores and gave comprehensibility (Fig 2) as well as perceived accentedness (Fig 3) ratings to eight sentence excerpts to understand their accent perception at a general level (*contra* specific segments as in Experiment 2 below). Female listeners (turquoise) appear to provide higher intelligibility (Fig 1) and comprehensibility (Fig 2) scores than their male counterparts. Those more satisfied with their own accents seem to give higher accentedness ratings (Fig 3). Summary statistics (S5 Table) appears to show that, among different accent types, the Standard American sample appears to yield higher intelligibility and comprehensibility and lower accentedness ratings, than moderately accented learner samples (i.e. ManMod and CanMod).

To determine if the participants rated the accent samples differently, we fitted cumulative link linear mixed-effects models to the three types of scaled results using the R package *ordinal* [39]. Models were constructed using a bottom-up approach using the *anova()* function. All three models included by-subject random intercepts and the fixed factors of ‘Accent’ and ‘Self-satisfaction’ (model summaries in S2 Table). The factor ‘Accent’ corresponds to the Mandarin, Cantonese and English L1 backgrounds and accent strength. Planned ‘Accent’ contrasts were defined to test H1-H3. The ‘Self-satisfaction’ factor refers to participants’ responses to the survey question Q1 (To what extent are you satisfied with your English Accent?). The models for accentedness and comprehensibility, the ‘Equal’ factor was also included. It refers to participants’ responses to Q6 (What is your opinion on the idea that all accents are equal in status?). In the models for intelligibility and comprehensibility, we also included the fixed factor ‘Gender’ as well as its interaction with Accent. In the model for comprehensibility, we further included the interaction term ‘Equal:Accent’. We used the *emmeans* package [40] for post-hoc comparisons to locate statistically significant differences where applicable. *emmeans()* uses the Tukey method to account for multiple comparisons.

In the model for intelligibility (see Fig 1), the main effects of self-satisfaction (*X*^*2*^(1) = 15.191, *p* < .001), gender (*Χ*^*2*^(1) = 5.897, *p* = .015), and accent (*Χ*^2^(7) = 44.511, *p* < .001) were significant, as was the interaction between accent and gender (*Χ*^2^(7) = 20.334, *p* = .005). Post-hoc comparisons showed that female participants gave significantly higher intelligibility ratings than male participants (*p* = .012). Those who were more positive towards their own accent (*β* = 1.462, *SE* = 0.370, *z* = 3.957, *p* < .001) gave significantly higher intelligibility ratings. The interaction between gender and accent reflects that the difference in intelligibility ratings between Mandarin and Cantonese samples was significantly larger for male participants in the strongly accented (*β* = 3.278, *SE* = 1.183, *z* = 2.770, *p* = .006) and moderately accented conditions (*β* = 1.855, *SE =* 0.831, z = 2.232, *p* = .026), while smaller for male participants in the weakly accented condition (*β* = -3.006, *SE* = 1.309, z = -2.296, *p* = .022). No other significant contrast was found.

In the model for comprehensibility (see Fig 2), the main effects of self-satisfaction (*Χ*^*2*^(1) = 17.724, *p* < .001), ‘Equal’ (*Χ*^*2*^(1) = 5.356, *p* = .021), gender (*Χ*^*2*^(1) = 6.915, *p* = .009), and accent (*Χ*^2^(7) = 59.841, *p* < .001) were significant, as were the interaction terms accent and gender (*Χ*^2^(7) = 17.677, *p* = .014) and between ‘Equal’ and accent (*Χ*^2^(7) = 17.951, *p* = .012). Those happy with their own accent tended to give higher comprehensibility ratings (*β* = 1.059, *SE* = 0.235, z = 4.509, *p* < .001). The main effect of accent was qualified by an interaction with gender. The relative ratings on Mandarin and Cantonese stimuli were in opposite directions for male vs. female participants in both the strongly accented (*β* = 2.516, *SE* = 1.112, *z* = 2.262, *p* = .024) and the weakly accented conditions (*β* = -3.420, *SE* = 1.232, *z* = -2.775, *p* = .006). The difference between Standard American and moderately accented stimuli was larger for female than male listeners (*β* = -3.308, *SE* = 1.366, z = -2.422, *p* = .015), whereas the opposite was true for the difference between strongly accented and weakly accented Mandarin stimuli (*β* = 3.838, *SE* = 1.593, *z* = 2.409, *p* = .016). Although those who believe that some accents are superior to others tended to give higher comprehensibility ratings (*β* = 0.424, *SE* = 0.181, *z* = 2.344, *p* = .019), this main effect was qualified by an interaction with accent. Compared with the weakly accent Cantonese stimulus, the weakly accented Mandarin stimuli saw the lowest comprehensibility ratings for those giving 3 or 4 to the ‘Equal’ question (*β* = 1.102, *SE* = 0.552, z = 1.996, *p* = .046). Compared with the Standard American stimulus, the moderately accented stimuli yielded the lowest comprehensibility ratings where listeners gave 4 to the ‘Equal’ question (*β* = 1.530, *SE* = 0.623, *z* = 2.457, *p* = .014); similarly, the strongly accented stimuli saw lower ratings given by listeners who responded 4 to the ‘Equal’ question.

In the accentedness data (see Fig 3), there were significant main effects of self-satisfaction (*Χ*^*2*^(1) = 15.681, *p* < .001), ‘Equal’ (*Χ*^*2*^(1) = 10.655, *p* = .001), and accent (*Χ*^2^(7) = 49.817, *p* < .001). Post-hoc comparisons confirmed that the Standard American accent sample was rated significantly lower in accentedness than the strongly accented (*p* = .012) and moderately accented learner samples (*p* < .001). Among the Mandarin-accented stimuli, the strongly accented stimulus was considered significantly more accented than the weakly accented stimulus (*p* < .001). No other significant contrast was found.

### Experiment 2

**Fig 4:**
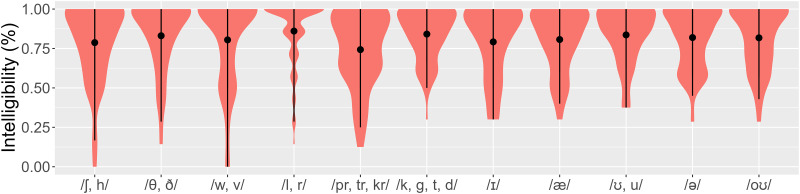
Intelligibility scores by segment type (dot = mean, interval = quantile).

Fig 4 shows intelligibility scores (% correct) by consonant and vowel types. For both consonants and vowels, the rightmost category represents sounds hypothesized to be less challenging. Compared with the baseline stop sounds /k, g, t, d/, fricatives /ʃ, h/ and the consonant clusters /pr, tr, kr/ appear to yield fewer correct responses. The intelligibility ratings for the vowels are relatively similar.

**Fig 5:**
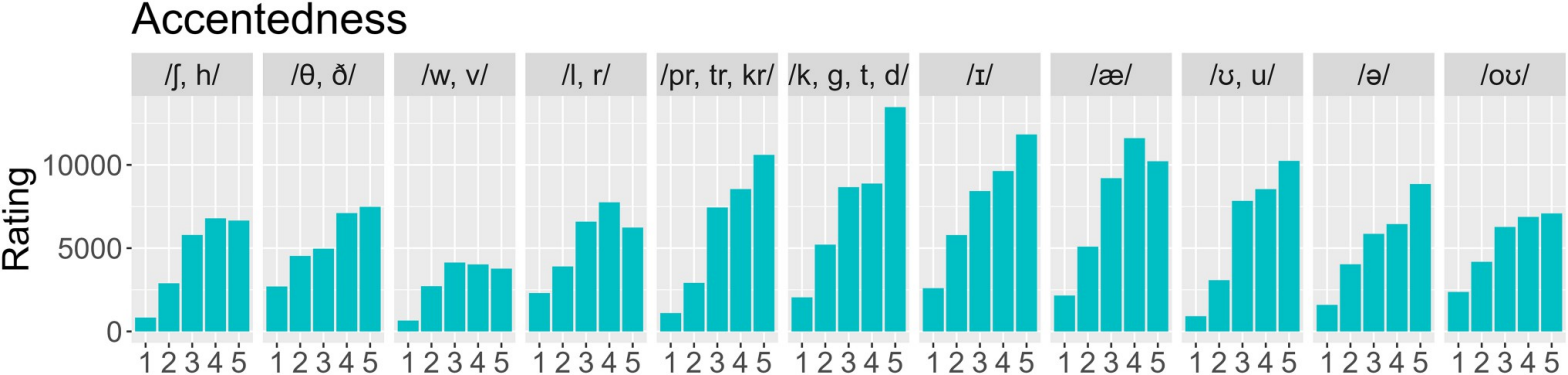
Distribution of accentedness ratings by segment type.

As for perceived accentedness, Fig 5 shows that the groups /θ, ð/, /w, v/, and /l, r/ all seem to yield lower accentedness ratings than the baseline stop sounds. Compared with the baseline diphthong /oʊ/, both /æ/ and /ʊ, u/ appear to be considered more accented. See S6 Table for more detailed summary statistics regarding Figs 4 and 5.

We fitted a logistic mixed effects model to the binary intelligibility data (correct/incorrect) (see S3 Table). The best-fitting model contained by-subject random intercepts as well as the fixed factor of challenging vs. non-challenging consonants (*Χ*^*2*^(5) = 48.08, *p* < .001). There was no other significant main effect. Post-hoc comparisons confirm that compared with the baseline stop sounds, both fricatives /ʃ, h/ (*p* = .004) and clusters /pr, tr, kr/ (*p* < .001) yielded significantly fewer correct responses. No other condition was found to be significantly different from the baseline.

We fitted another cumulative link linear mixed effects model to the accent rating data (see S3 Table). The best-fitting model contained by-subject random intercepts and random slopes for consonants, as well as the fixed factors of the consonants (*Χ*^*2*^(5) = 81.958, *p* < .001), vowels (*Χ*^*2*^(4) = 16.305, *p* = .003), their interaction (*Χ*^*2*^(19) = 289.710, *p* < .001), as well as word length (i.e. number of syllables) (*Χ*^*2*^(1) = 18.676, *p* < .001).

For the consonants, compared with the baseline level, participants gave significantly lower accentedness ratings to words starting with the consonants /θ, ð/ (*β* = -1.504, *SE* = 0.260, *z* = -5.777, *p* < .001), /w, v/ (*β* = -0.967, *SE* = 0.394, *z* = -2.456, *p* = .014), and /l, r/ (*β* = -0.892, *SE* = 0.227, *z* = -3.935, *p* < .001), but higher to those starting with a consonant cluster (*β* = 0.837, *SE* = 0.256, *z* = 3.270, *p* = .001). For the vowels, compared with the baseline level /oʊ/, participants gave significantly higher perceived accentedness ratings in the condition /ə/ (*β* = 2.620, *SE* = 0.362, *z* = 7.228, *p* < .001) and lower ratings in the condition /ɪ/ (*β* = -0.745, *SE* = 0.203, *z* = -3.659, *p* < .001). A longer word is associated with lower accent ratings (*β* = -1.268, *SE* = 0.289, *z* = -4.380, *p* < .001). The main effect of consonants is also qualified by an interaction with vowels (see S3 Table). This interaction is illustrated in S4 Table: for various vowel conditions, certain consonant conditions (underlined) yielded anomalously strong/weak accentedness ratings (compared with the baseline /oʊ/). For example, the difference between /θ, ð/ and /k, g, t, d/ is significantly larger for /æ/ than for baseline (*β* = 1.779, *SE* = 0.319, *z* = 5.586, *p* < .001), and likewise for /ʊ, u/ (*β* = 2.027, *SE* = 0.357, *z* = 5.672, *p* < .001). Interestingly, the more intelligible sounds to learners were those which have been documented as “challenging” in the previous literature [31, 32], namely words with /θ, ð/, /w, v/ and /l, r/. Moreover, words with /θ, ð/, /w, v/ and /l, r/ were rated less accented than the cluster /pr, tr, kr/ and fricatives /ʃ, h/. However, the baseline control sounds /b, k, g, t, d/ were rated as highly intelligible and highly accented. As for the vowels, /æ/ and /ʊ, u/ received high accentedness ratings without showing lower intelligibility, which differs from Deterding’s [32] findings that Mandarin speakers are insensitive to the difference between /u/ and /ʊ/.

## Discussion

The overall findings demonstrate an apparent mismatch among intelligibility, comprehensibility, and perceived accentedness, which echoes the contention by Gluszek et al. [41] that subjective and objective comprehensibility are fundamentally different. Experiment 1 results supported neither H1 nor H2 since both the intelligibility and comprehensibility scores of Standard American English speech outperformed those of Chinese speech. The results only partly supported H3 as Mandarin speakers were rated the most accented, but only in the strong-accent condition. The coexistence of high intelligibility and high accentedness predicted by H4 was supported. Experiment 2 results refuted H5 as the challenging sounds yielded neither lower intelligibility nor higher accentedness than the non-challenging ones. The survey results implied that Chinese learners of English might relate a strong learner accent to a lack of confidence, low superiority, and lack of in-group loyalty. From the findings, we contend that native-speakerism still prevails in China.

### H1: Intelligibility

The overall postulation of H1 (Mandarin-accented speech would yield the highest overall intelligibility) was refuted by higher intelligibility scores of Standard American English stimuli; the strength-wise postulation of H1 (intelligibility patterns of Cantonese and Mandarin speech would be the opposite) was refuted by higher intelligibility scores for both Cantonese and Mandarin weak-accent speech than for the stronger ones. The familiar Mandarin accent was still favored over Cantonese because the Mandarin stimuli were better understood, as reflected by higher intelligibility scores (See Fig 1). While Mandarin speakers tend to regard Mandarin speech as more intelligible than Cantonese, the opposite was true for British or Standard American English listeners, as shown in a previous study [12]. Overall, the findings on intelligibility are not in line with previous studies [22, 23] claiming that the L2 learner’s own variety of English was better understood than the British or Standard American English varieties. The familiarity advantage seems to exist only in the Cantonese-Mandarin contrast in the current investigation. One conceivable explanation could be the proximity effect, because people who are familiar with their own regional accent may understand the stimuli more easily (we owe this interpretation to Reviewer 1).

### H2: Comprehensibility

Contrary to previous claims [22, 24], L2 Mandarin listeners in Experiment 1 did not find English spoken by Mandarin speakers to have higher comprehensibility scores than that of Standard American English speakers. As the overall comprehensibility ratings of Mandarin speech were higher than those of Cantonese, thus H2 (Mandarin-accented speech showing the lowest comprehensibility) was also not supported. However, in terms of accent strengths, the Mandarin weak-accent stimuli were consistently rated as more comprehensible than the stronger ones. In contrast, the comprehensibility ratings of Cantonese stimuli did not follow this pattern. Cantonese-accented English as an unfamiliar variety was more difficult for Mandarin speakers to understand than Mandarin-accented English, even in the weakly accented condition. It suggests that the L1 familiarity advantage does play a role in the subjective perception of accented English.

### H3: Accentedness

The Mandarin-accented stimuli were rated lower than the Cantonese-accented ones in terms of overall accentedness, which refuted H3 (Mandarin stimuli would be the most accented). Although English speakers were placed as the least accented among the three speaker groups, the perceived accent level of the Standard American English variant was still high, suggesting that listeners might take the standard accent as foreign-accented, too.

However, H3’s prediction was partly supported in the strong-accent condition. Interestingly, the Cantonese moderate-accent stimulus was rated more accented than the other two Cantonese conditions. In other words, the stimuli with stronger Cantonese accents were rated neither consistently higher nor lower than weaker ones, suggesting that accent strength might not always reliably match L2 listeners’ beliefs. This affirms previous findings that intelligibility and comprehensibility are not always in parallel [24].

### H4: Bias towards L2 accents

Considering the intelligibility and accentedness results altogether, we would find that the high perceived accentedness (3.444) scores coexisted with high intelligibility (3.519) scores for the moderate-accent Mandarin stimuli. Moreover, although two-tailed paired samples t-tests showed that accentedness and intelligibility ratings were significantly different for Cantonese- (*MD* = -0.259, *p* = .020), Mandarin- (*MD* = -0.454, *p* = < .001), and Standard American English speech (*MD* = -0.870, *p* < .001), the mean difference for the latter was the largest. Since a larger difference indicates better intelligibility and a weaker accent, H4 was supported. Given that good intelligibility coupled with high accentedness may lead to unfair accent evaluations [23, 24], this mismatch may reflect learners’ harsh evaluations of Chinese-accented L2 English sounds despite good understanding. In other words, the high perceived accentedness and high actual intelligibility of Mandarin accented English indicates that L2 speakers were prone to be more aware of the L2 accents than of the Standard American variety.

### H5: Individual words

In Experiment 2, we found that the same words associated with high intelligibility were also linked to high perceived accentedness, thus not confirming H5, which postulated that the words containing challenging consonants and vowels [31, 32] would yield lower levels of intelligibility and higher levels of accentedness than those containing non-challenging ones. Again, this mismatch suggests high awareness in the evaluation of Chinese-accented English.

### Native-favoring and self-mocking stereotypes

The survey results show that many learners are unconfident about their accent for fear of low superiority, coinciding with Pan and Block [42]’s report that Chinese ESL learners’ endorsing a “native-like” English pronunciation may have led to learners’ lack of confidence in their own accent. Therefore, the bias rooted in native-speakerism [10, 14] still exists among Chinese ESL learners. Evidence elsewhere includes Austrian learners of English who assigned a lower status to their own accent than other varieties of English [43] and Japanese learners of English believing their Japanese-accented English was inferior to Malaysian and Korean accents of English [44].

Moreover, previous studies reported that listeners might locate incoming L2 speech on a scale of status-stressing vs. solidarity-stressing norms [45], such as whether listeners prefer their own familiar accents, which enhance feelings of in-group solidarity over other varieties. For example, Giles and Edwards [46] reported that previous in-group members who try to adopt a standard variety might risk being marginalized as a “sell-out” by their speech community; McKenzie and Gilmore [47] found high in-group loyalties in Japanese ESL learners’ self-evaluations of their English accent. The negative comments on regional accents shown in Q3 (accent-related comments), however, suggest a lack of in-group loyalty among Chinese learners of English: the consistently lower comprehensibility ratings on strong-accent learner speech indicate that the L2 speakers who approximated the Standard American variety were favored, instead of being considered as a “sell-out” or “betrayer”, by the listeners. We may conclude that the paradigm shift from native-speakerism to world Englishes in China still awaits, but we may do the following to change the status quo. (a) As Munro [7] and He and Li [12] suggested, language policymakers and education specialists should not refer to learners’ L2 regional accents as “non-standard”, “inaccurate”, or “not ‘native’ enough”. (b) Learners should be informed to set reasonable learning goals in pronunciation and to stop employing a (self-)mocking attitude towards speakers with a non-mainstream regional accent.

### Limitations and prospects

The experimental design used in the current study had a few limitations. Firstly, studies on language attitudes tend to establish accent categorization with larger samples of ratings or through a larger focus group of expert raters to avoid impressionistic judgments [48]. However, due to limited time and funding, we only managed to recruit two expert judges for categorization. Secondly, to avoid causing participants excessive fatigue, the stimuli covered only the selected vowels and consonants at the expense of comprehensiveness. Thirdly, since the binary data did not distinguish whether the consonant or vowel was (mis)heard by the participants, the results of Experiment 2 only allow us to elicit the intelligibility and accentedness for the words containing particular consonants and vowels, but not for the sounds themselves. In addition, some participants (N = 11) were bilingual in Mandarin and Cantonese–arguably a potential confound. However, this combination of language backgrounds has the advantage of matching China’s demographics well (According to the Steering Group Office for Survey of Language Situation in China [49], about 19.2% of the population in Guangdong province were bilinguals in Mandarin and Cantonese). Along with the assumption that members of the same speech community would have shared language attitudes and norms, we decided against further restricting participants’ L1 backgrounds.

Future studies may collect Chinese English accent perception data from Standard American English speakers and compare those with the present ones to shed more light on the discrimination of accents [41]. Future studies may also examine the possible accent-related biases in spoken English tests, especially those administered by L2-speaking examiners [50].

## Supporting information

S1 Appendix Links to stimulus excerpts from the speech accent archive. (DOCX)

S2 Appendix Experiment 1 instructions. (DOCX)

S1 Table Responses to Q2 and Q3. (DOCX)

S2 Table Experiment 1 model summaries. (DOCX)

S3 Table Experiment 2 model summaries. (DOCX)

S4 Table Mean accentedness ratings by consonant and vowel conditions. (DOCX)

S5 Table Summary statistics of Experiment 1 rating results (as continuous data). (DOCX)

S6 Table Summary statistics of Experiment 2 results (as continuous data). (DOCX)
