# Supplementary material for: Portraying accent stereotyping by second language speakers
Source: PLoS One. 2023 Jun 15;18(6):e0287172. doi: 10.1371/journal.pone.0287172 (PMC10270356; doi:10.1371/journal.pone.0287172)
Supplement: S1 Appendix — (DOCX) [file pone.0287172.s001.docx]

**Supporting information**

**S1 Appendix. Links to stimulus excerpts from the *Speech Accent Archive***

Excerpt 1: Cantonese1, female, Hong Kong, China

http://accent.gmu.edu/browse_language.php?function=detail&speakerid=45

Excerpt 2: Mandarin33, male, Beijing, China

http://accent.gmu.edu/browse_language.php?function=detail&speakerid=1541

Excerpt 3: Cantonese9, male, Hong Kong, China

http://accent.gmu.edu/browse_language.php?function=detail&speakerid=502

Excerpt 4: Mandarin118, female, Xi’an, Shaanxi, China

http://accent.gmu.edu/browse_language.php?function=detail&speakerid=2679

Excerpt 5: Mandarin87, female, Lanzhou, Gansu, China

http://accent.gmu.edu/browse_language.php?function=detail&speakerid=2435

Excerpt 6: Mandarin139, male, Kunming, Yunnan, China

http://accent.gmu.edu/browse_language.php?function=detail&speakerid=2882

Excerpt 7: Cantonese2, male, Hong Kong, China

http://accent.gmu.edu/browse_language.php?function=detail&speakerid=46

Excerpt 8: English33, male, San Diego, California, USA

http://accent.gmu.edu/browse_language.php?function=detail&speakerid=92
