# Supplementary material for: Portraying accent stereotyping by second language speakers
Source: PLoS One. 2023 Jun 15;18(6):e0287172. doi: 10.1371/journal.pone.0287172 (PMC10270356; doi:10.1371/journal.pone.0287172)
Supplement: S2 Appendix — (DOCX) [file pone.0287172.s002.docx]

**Supporting information**

**S2 Appendix. Experiment 1 Instructions**

| 实验一说明 Experiment 1 Instructions  听下列8段声音，每段结束后您有半分钟时间完成以下三个任务。  (Listen to the following 8 paragraphs and complete the three tasks below after each paragraph. You have 30 seconds.)  任务1. 回答下列问题(例)：  (Task 1: Example: answer the following questions briefly.)  Who was called?  What was bought from the store?  What was bought for Bob?  Where were they going to meet?  任务2. 你同意以下说法吗？完全不同意=1，完全同意=5。(Task 2: Do you agree the following statement? give numbers of 1 (strongly disagree) to 5 (strongly agree))  我不需要费力才能听懂这一段英文内容。1 2 3 4 5  (I don’t have to make an effort to understand the excerpt.) |
| --- |
| 任务3. 你同意以下说法吗？完全不同意=1，完全同意=5。 (Task 3: Do you agree the following statement? give numbers of 1 (strongly disagree) to 5 (strongly agree))  这段英文内容的口音很重。1 2 3 4 5  (The excerpt is heavily accented.) |
