# Supplementary material for: Portraying accent stereotyping by second language speakers
Source: PLoS One. 2023 Jun 15;18(6):e0287172. doi: 10.1371/journal.pone.0287172 (PMC10270356; doi:10.1371/journal.pone.0287172)
Supplement: S1 Table — (DOCX) [file pone.0287172.s003.docx]

**Supporting information**

**S3 Table. Responses to Q2 and Q3.**

Q2: Familiar varieties of English

| ***Frequently mentioned*** | British (39) (Scottish, Welsh, SSBE), Canadian, Australian, | American (33) (Standard American, Southern American, “country English”, Texas) | Chinese (29) (Guangzhou Cantonese, Chaoshan, Hakka, Hong Kong Cantonese) | Indian (20) | Japanese (18) | Irish (14) |
| --- | --- | --- | --- | --- | --- | --- |
| ***Spotted*** | Other European countries: French (3), German (1) | | | | | |
|  | Other Asian countries: Korean (4), Singaporean (3), Sri Lankan (1), Malay (1), Vietnamese (1), Filipino (1) | | | | | |

Q3: Recurring comments received on “non-mainstream” regional English accents

| ***Enunciation and fluency*** | ***Prosody*** | ***Emotive evaluations*** |
| --- | --- | --- |
| intelligibility impeded, too fast or disfluent, too many pauses, going on and on without stopping | no ups and downs; improper rhythm or pauses | annoyingly unable to understand, (especially Northeastern China or Xinjiang) |
| problems with specific sounds (e.g., nasals, voiced consonants) | unnatural intonation or lack of variation in intonation | sounds too formal, pretentious, outdated, unattractive (Northeastern China) |
| isolated sounds without linking, deletion of specific sounds | lack of emotion | associated with low social status, corny (especially Northeastern China) |
| vowels not fully pronounced | improper rhythm and pitch | sounds funny (Cantonese), non-standard (Northern) |
