# Supplementary material for: Portraying accent stereotyping by second language speakers
Source: PLoS One. 2023 Jun 15;18(6):e0287172. doi: 10.1371/journal.pone.0287172 (PMC10270356; doi:10.1371/journal.pone.0287172)
Supplement: S2 Table — (DOCX) [file pone.0287172.s004.docx]

**Supporting information**

**S4 Table. Experiment 1 model summaries**

|  | **Intelligibility** | | | | **Perceived accentedness** | | | | **Comprehensibility** | | | |
| --- | --- | --- | --- | --- | --- | --- | --- | --- | --- | --- | --- | --- |
|  | *β* | *SE* | *z* | *Pr(>\|z\|)* | *β* | *SE* | *z* | *Pr(>\|z\|)* | *β* | *SE* | *z* | *Pr(>\|z\|)* |
| Accent_(Native-Strong)_ | -1.953 | 1.093 | -1.787 | .074 | **2.093** | **0.963** | **2.174** | **.030** | 4.337 | 3.217 | 1.348 | .178 |
| Accent_(CanStrong-ManStrong)_ | **-1.499** | **0.590** | **-2.539** | **.011** | **1.374** | **0.516** | **2.663** | **.008** | 1.103 | 1.734 | 0.636 | .525 |
| Accent_(Native-Mod)_ | **3.329** | **0.741** | **4.492** | **< .001** | **-2.956** | **0.645** | **-4.584** | **< .001** | -1.579 | 2.126 | -0.743 | .458 |
| Accent_(CanMod-ManMod)_ | **-0.847** | **0.415** | **-2.041** | **.041** | 0.451 | 0.364 | 1.240 | .215 | 1.291 | 1.226 | 1.053 | .292 |
| Accent_(Native-Weak)_ | -0.190 | 1.145 | -0.166 | .868 | -0.290 | 1.010 | -.287 | .774 | -3.945 | 3.351 | -1.177 | .239 |
| Accent_(CanWeak-ManWeak)_ | **2.304** | **0.662** | **3.484** | **< .001** | -0.513 | 0.568 | -.904 | .366 | -1.440 | 1.871 | -0.769 | .442 |
| Accent_(ManStrong-ManWeak)_ | **-3.200** | **0.857** | **-3.736** | **< .001** | **3.010** | **0.737** | **4.082** | **< .001** | -0.212 | 2.447 | -0.087 | .931 |
| SelfSatis | **1.462** | **0.370** | **3.957** | **< .001** | **0.845** | **0.203** | **4.161** | **< .001** | **1.059** | **0.235** | **4.509** | **< .001** |
| Equal |  |  |  |  | **0.557** | **0.160** | **3.487** | **< .001** | **0.424** | **0.181** | **2.344** | **.019** |
| Gender | **-1.596** | **0.632** | **-2.526** | **.012** |  |  |  |  | **-1.333** | **0.408** | **-3.267** | **.001** |
| Gender:Accent_(Native-Strong)_ | 1.402 | 2.175 | 0.645 | .519 |  |  |  |  | 2.909 | 2.061 | 1.412 | .158 |
| Gender:Accent_(CanStrong-ManStrong)_ | **3.278** | **1.183** | **2.770** | **.006** |  |  |  |  | **2.516** | **1.112** | **2.262** | **.024** |
| Gender:Accent_(Native-Mod)_ | -1.869 | 1.442 | -1.296 | .195 |  |  |  |  | **-3.308** | **1.366** | **-2.422** | **.015** |
| Gender:Accent_(CanMod-ManMod)_ | **1.855** | **0.831** | **2.232** | **.026** |  |  |  |  | 0.430 | 0.775 | 0.555 | .579 |
| Gender:Accent_(Native-Weak)_ | -1.186 | 2.292 | -0.517 | .605 |  |  |  |  | -0.245 | 2.167 | -0.113 | .910 |
| Gender:Accent_(CanWeak-ManWeak)_ | **-3.006** | **1.309** | **-2.296** | **.022** |  |  |  |  | **-3.420** | **1.232** | **-2.775** | **.006** |
| Gender:Accent_(ManStrong-ManWeak)_ | 2.964 | 1.684 | 1.760 | .078 |  |  |  |  | **3.838** | **1.593** | **2.409** | **.016** |
| Equal:Accent_(Native-Strong)_ |  |  |  |  |  |  |  |  | **-2.111** | **0.951** | **-2.219** | **.026** |
| Equal:Accent_(CanStrong-ManStrong)_ |  |  |  |  |  |  |  |  | -0.966 | 0.513 | -1.882 | .060 |
| Equal:Accent_(Native-Mod)_ |  |  |  |  |  |  |  |  | **1.530** | **0.623** | **2.457** | **.014** |
| Equal:Accent_(CanMod-ManMod)_ |  |  |  |  |  |  |  |  | **-0.723** | **0.357** | **-2.028** | **.043** |
| Equal:Accent_(Native-Weak)_ |  |  |  |  |  |  |  |  | 1.528 | 0.988 | 1.546 | .122 |
| Equal:Accent_(CanWeak-ManWeak)_ |  |  |  |  |  |  |  |  | **1.102** | **0.552** | **1.996** | **.046** |
| Equal:Accent_(ManStrong-ManWeak)_ |  |  |  |  |  |  |  |  | -1.176 | 0.726 | -1.620 | .105 |
