# Supplementary material for: Portraying accent stereotyping by second language speakers
Source: PLoS One. 2023 Jun 15;18(6):e0287172. doi: 10.1371/journal.pone.0287172 (PMC10270356; doi:10.1371/journal.pone.0287172)
Supplement: S3 Table — (DOCX) [file pone.0287172.s005.docx]

**Supporting information**

**S5 Table. Experiment 2 model summaries**

|  | **Intelligibility** | | | | **Perceived accentedness** | | | |
| --- | --- | --- | --- | --- | --- | --- | --- | --- |
|  | *β* | *SE* | *z* | *Pr(>\|z\|)* | *β* | *SE* | *z* | *Pr(>\|z\|)* |
| (Intercept) | 2.242 | 0.189 | 11.862 | < .001 |  |  |  |  |
| Consonant /ʃ, h/ | **-0.442** | **0.155** | **-2.850** | **.004** | 0.366 | 0.253 | 1.443 | .149 |
| Consonant /θ, ð/ | -0.093 | 0.155 | -0.601 | .548 | **-1.504** | **0.260** | **-5.777** | **< .001** |
| Consonant /w, v/ | -0.310 | 0.179 | -1.727 | .084 | **-0.967** | **0.394** | **-2.456** | **.014** |
| Consonant /l, r/ | 0.179 | 0.161 | 1.117 | .264 | **-0.892** | **0.227** | **-3.935** | **< .001** |
| Consonant /pr, tr, kr/ | **-0.760** | **0.140** | **-5.414** | **< .001** | **0.837** | **0.256** | **3.270** | **.001** |
| Vowel /ɪ/ |  |  |  |  | **-0.744** | **0.203** | **-3.659** | **< .001** |
| Vowel /æ/ |  |  |  |  | 0.061 | 0.202 | 0.303 | .762 |
| Vowel /ʊ, u/ |  |  |  |  | 0.089 | 0.203 | 0.436 | .663 |
| Vowel /ə |  |  |  |  | **2.620** | **0.362** | **7.228** | **< .001** |
| Word length |  |  |  |  | **-1.268** | **0.289** | **-4.380** | **< .001** |
| Consonant /ʃ, h/ :Vowel /ɪ/ |  |  |  |  | **0.687** | **0.317** | **2.165** | **.030** |
| Consonant /θ, ð/:Vowel /ɪ/ |  |  |  |  | **1.502** | **0.318** | **4.721** | **< .001** |
| Consonant /w, v/ :Vowel /ɪ/ |  |  |  |  | **2.521** | **0.354** | **7.115** | **< .001** |
| Consonant /l, r/ :Vowel /ɪ/ |  |  |  |  | **2.669** | **0.335** | **7.959** | **< .001** |
| Consonant /pr, tr, kr/:Vowel /ɪ/ |  |  |  |  | **0.831** | **0.361** | **2.304** | **.021** |
| Consonant /ʃ, h/ :Vowel /æ/ |  |  |  |  | -0.418 | 0.346 | -1.208 | .227 |
| Consonant /θ, ð/:Vowel /æ/ |  |  |  |  | **1.779** | **0.319** | **5.586** | **< .001** |
| Consonant /w, v/ :Vowel /æ/ |  |  |  |  | **0.948** | **0.452** | **2.096** | **.036** |
| Consonant /l, r/ :Vowel /æ/ |  |  |  |  | 0.021 | 0.281 | 0.075 | .940 |
| Consonant /pr, tr, kr/:Vowel /æ/ |  |  |  |  | **-0.963** | **0.323** | **-2.983** | **.003** |
| Consonant /ʃ, h/ :Vowel /ʊ, u/ |  |  |  |  | 0.159 | 0.349 | 0.456 | .648 |
| Consonant /θ, ð/:Vowel /ʊ, u/ |  |  |  |  | **2.027** | **0.357** | **5.672** | **< .001** |
| Consonant /w, v/ :Vowel /ʊ, u/ |  |  |  |  | 0.721 | 0.452 | 1.594 | .111 |
| Consonant /l, r/ :Vowel /ʊ, u/ |  |  |  |  | 0.555 | 0.311 | 1.784 | .074 |
| Consonant /pr, tr, kr/:Vowel /ʊ, u/ |  |  |  |  | **-0.647** | **0.323** | **-2.003** | **.045** |
| Consonant /ʃ, h/ :Vowel /ə/ |  |  |  |  | **-3.907** | **0.461** | **-8.472** | **< .001** |
| Consonant /θ, ð/:Vowel /ə/ |  |  |  |  | **-2.341** | **0.461** | **-5.080** | **< .001** |
| Consonant /l, r/ :Vowel /ə/ |  |  |  |  | **-1.403** | **0.324** | **-4.327** | **< .001** |
| Consonant /pr, tr, kr/:Vowel /ə/ |  |  |  |  | **-1.754** | **0.366** | **-4.797** | **< .001** |

Baseline levels: consonant /b, k, g, t, d/, vowel /oʊ/. Follow-up analyses with the bilingual speakers’ data removed did not yield different results.
