# Supplementary material for: Portraying accent stereotyping by second language speakers
Source: PLoS One. 2023 Jun 15;18(6):e0287172. doi: 10.1371/journal.pone.0287172 (PMC10270356; doi:10.1371/journal.pone.0287172)
Supplement: S4 Table — (DOCX) [file pone.0287172.s006.docx]

**Supporting information**

**S6 Table. Mean accentedness ratings by consonant and vowel conditions**

For each vowel (bar baseline condition), one consonant condition with anomalous accentedness ratings is underlined.

|  | /ɪ/ | | /æ/ | | /ʊ, u/ | | /ə/ | | /əu/ | | **Total** | |
| --- | --- | --- | --- | --- | --- | --- | --- | --- | --- | --- | --- | --- |
|  | M | SD | M | SD | M | SD | M | SD | M | SD | M | SD |
| /ʃ, h/ | 3.9 | 1.1 | 3.7 | 1.2 | 4.0 | 1.1 | 3.2 | 1.2 | 3.9 | 1.1 | 3.8 | 1.1 |
| /θ, ð/ | 3.3 | 1.3 | **3.9** | 1.1 | **4.0** | 1.2 | 3.0 | 1.4 | 2.9 | 1.3 | 3.5 | 1.3 |
| /w, v/ | 3.4 | 1.3 | 3.7 | 1.1 | 3.7 | 1.1 | 3.2 | 1.1 | N/A | | 3.5 | 1.2 |
| /l, r/ | 4.2 | 1.1 | 3.3 | 1.2 | 3.6 | 1.0 | 3.2 | 1.3 | 3.2 | 1.3 | 3.4 | 1.3 |
| Clusters | 3.8 | 1.1 | 3.7 | 1.2 | 3.9 | 1.0 | 3.9 | 1.1 | 4.2 | 0.9 | 3.9 | 1.1 |
| /k, g, t, d/ | **3.3** | 1.3 | 3.7 | 1.2 | 3.7 | 1.2 | **4.3** | 1.0 | 3.7 | 1.2 | 3.8 | 1.2 |
| **Total** | 3.6 | 1.3 | 3.7 | 1.2 | 3.8 | 1.1 | 3.6 | 1.3 | 3.5 | 1.3 | 3.7 | 1.2 |
