# Supplementary material for: Portraying accent stereotyping by second language speakers
Source: PLoS One. 2023 Jun 15;18(6):e0287172. doi: 10.1371/journal.pone.0287172 (PMC10270356; doi:10.1371/journal.pone.0287172)
Supplement: S5 Table — (DOCX) [file pone.0287172.s007.docx]

**Supporting information**

**S7 Table. Summary statistics of Experiment 1 rating results (as continuous data)**

|  |  |  | Intelligibility | | Comprehensibility | | Accentedness | |
| --- | --- | --- | --- | --- | --- | --- | --- | --- |
|  |  |  | *M* | *SD* | *M* | *SD* | *M* | *SD* |
| **Male** | SelfSatis ≤2 | CanMod | 2.8 | 1.1 | 2.3 | 0.9 | 3.4 | 1.0 |
|  |  | CanStrg | 3.3 | 1.1 | 2.8 | 1.0 | 2.7 | 1.3 |
|  |  | CanWk | 3.3 | 1.4 | 2.9 | 1.2 | 3.0 | 1.3 |
|  |  | ManMod | 3.0 | 1.0 | 2.9 | 0.9 | 2.7 | 1.1 |
|  |  | ManStrg | 2.9 | 0.8 | 2.8 | 1.0 | 3.0 | 1.3 |
|  |  | ManWk | 3.3 | 1.2 | 3.2 | 1.1 | 2.8 | 1.2 |
|  |  | Native | 3.3 | 1.2 | 3.4 | 1.1 | 2.6 | 1.0 |
|  |  | ≤2 | 3.2 | 1.1 | 2.9 | 1.1 | 2.9 | 1.2 |
|  | SelfSatis >2 | CanMod | 3.6 | 0.8 | 3.3 | 0.9 | 3.7 | 0.7 |
|  |  | CanStrg | 3.7 | 0.7 | 3.4 | 0.8 | 3.4 | 0.8 |
|  |  | CanWk | 3.8 | 0.8 | 3.5 | 0.8 | 3.2 | 0.8 |
|  |  | ManMod | 3.4 | 0.8 | 3.4 | 0.9 | 3.5 | 0.8 |
|  |  | ManStrg | 3.5 | 0.8 | 3.3 | 0.9 | 3.6 | 0.9 |
|  |  | ManWk | 3.7 | 0.8 | 3.4 | 0.8 | 3.2 | 0.9 |
|  |  | Native | 3.6 | 0.9 | 3.6 | 0.8 | 3.3 | 1.0 |
|  |  | >2 | 3.6 | 0.8 | 3.4 | 0.8 | 3.4 | 0.9 |
|  | **Male** | | **3.4** | **1.0** | **3.2** | **0.9** | **3.2** | **1.0** |
| **Female** | SelfSatis ≤2 | CanMod | 3.3 | 0.8 | 3.0 | 0.6 | 3.6 | 0.8 |
|  |  | CanStrg | 3.9 | 0.4 | 3.6 | 0.5 | 3.1 | 0.7 |
|  |  | CanWk | 4.3 | 0.5 | 4.0 | 0.0 | 2.6 | 1.0 |
|  |  | ManMod | 3.7 | 0.5 | 3.4 | 0.5 | 3.9 | 0.4 |
|  |  | ManStrg | 3.9 | 0.4 | 3.6 | 0.5 | 3.3 | 0.8 |
|  |  | ManWk | 3.9 | 0.5 | 3.6 | 0.8 | 2.4 | 1.0 |
|  |  | Native | 4.1 | 0.4 | 4.0 | 0.6 | 2.3 | 1.0 |
|  |  | ≤2 | 3.9 | 0.6 | 3.6 | 0.7 | 2.9 | 1.0 |
|  | SelfSatis >2 | CanMod | 3.4 | 1.1 | 3.1 | 1.3 | 3.8 | 1.0 |
|  |  | CanStrg | 3.6 | 1.0 | 3.5 | 0.8 | 3.6 | 1.1 |
|  |  | CanWk | 4.4 | 0.7 | 4.3 | 0.7 | 3.3 | 1.4 |
|  |  | ManMod | 3.9 | 1.0 | 3.6 | 1.0 | 3.8 | 1.0 |
|  |  | ManStrg | 3.9 | 0.8 | 3.6 | 0.9 | 3.4 | 0.6 |
|  |  | ManWk | 4.0 | 0.8 | 3.9 | 0.9 | 3.0 | 1.2 |
|  |  | Native | 4.2 | 0.8 | 4.1 | 0.7 | 3.0 | 1.2 |
|  |  | >2 | 3.9 | 0.9 | 3.7 | 0.9 | 3.4 | 1.1 |
|  | **Female** | | **3.9** | **0.8** | **3.7** | **0.9** | **3.2** | **1.1** |
| **Grand** | | | **3.6** | **0.9** | **3.4** | **0.9** | **3.2** | **1.1** |
