# Supplementary material for: Portraying accent stereotyping by second language speakers
Source: PLoS One. 2023 Jun 15;18(6):e0287172. doi: 10.1371/journal.pone.0287172 (PMC10270356; doi:10.1371/journal.pone.0287172)
Supplement: S6 Table — (DOCX) [file pone.0287172.s008.docx]

**Supporting information**

**S8 Table. Summary statistics of Experiment 2 results (as continue data)**

|  | Intelligibility (%) | | Accentedness (1-5) | |
| --- | --- | --- | --- | --- |
|  | *M* | *SD* | *M* | *SD* |
| /ʃ, h/ | 78.7 | 25.0 | 3.77 | 1.14 |
| /θ, ð/ | 83.1 | 22.3 | 3.47 | 1.33 |
| /w, v/ | 80.5 | 28.6 | 3.51 | 1.18 |
| /l, r/ | 86.0 | 20.7 | 3.43 | 1.25 |
| /pr, tr, kr/ | 74.3 | 24.0 | 3.86 | 1.10 |
| /k, g, t, d/ | 84.1 | 16.2 | 3.75 | 1.22 |
| /ɪ/ | 79.2 | 22.5 | 3.62 | 1.25 |
| /æ/ | 80.7 | 19.8 | 3.66 | 1.18 |
| /ʊ, u/ | 83.6 | 18.0 | 3.82 | 1.12 |
| /ə/ | 81.9 | 19.0 | 3.60 | 1.28 |
| /oʊ/ | 81.8 | 19.8 | 3.54 | 1.27 |
| **Grand Total** | **81.3** | **21.8** | **3.65** | **1.22** |
